# Sporotrichoid Keratoacanthomas: Case Report and Review of Neoplasms Presenting in a Sporotrichoid Pattern

**DOI:** 10.7759/cureus.3196

**Published:** 2018-08-23

**Authors:** Boya Abudu, Philip R Cohen

**Affiliations:** 1 School of Medicine, University of California San Diego, La Jolla, USA; 2 Dermatologist, San Diego Family Dermatology, San Diego, USA

**Keywords:** cancer, infection, keratoacanthoma, neoplasm, sporotrichoid, sporotrichosis, tumor

## Abstract

Sporotrichosis is a fungal infection known for its distinct pattern of infectious skin nodules. Several conditions can present with lesions that appear in a sporotrichoid pattern. An 82-year-old man that presented with three cutaneous nodules on his right leg in a sporotrichoid manner is described; biopsy of each lesion revealed a keratoacanthoma. In addition to keratoacanthomas, other neoplasms—albeit rarely—may be observed to occur in a sporotrichoid manner. These included squamous cell carcinoma (three patients), lymphoma (two patients), and one patient with each of the following: epithelioid sarcoma, Langerhans cell histiocytosis, melanoma, and peripheral nerve sheath tumor. The 10 patients whose cancer had cutaneous lesions that presented in a sporotrichoid distribution ranged from 28 to 83 years old. The tumors equally appeared on either the upper extremity (five patients) or the lower extremity (five patients). Treatments included systemic chemotherapy, surgical intervention, and radiation. Three of the patients died secondary to their tumors. In conclusion, various infections and some miscellaneous disorders can present in a sporotrichoid pattern. Keratoacanthomas can be added to the list of cancers (which include squamous cell carcinoma, lymphoma, epithelioid sarcoma, Langerhans cell histiocytosis, melanoma, and peripheral nerve sheath tumor) whose skin lesions have appeared in a sporotrichoid distribution. When cutaneous lesions appear in a sporotrichoid manner, biopsy of the tissue—for not only microscopic examination but also bacterial, fungal, and mycobacterial cultures—should be considered.

## Introduction

Keratoacanthomas are malignant skin neoplasms that may appear suddenly and grow rapidly [1,2]. Sporotrichosis is a fungal infection in which cutaneous lesions may demonstrate a lymphatic distribution; several other conditions have also been observed that demonstrate lesions with a sporotrichoid pattern [2,3]. A man with eruptive keratoacanthomas in a sporotrichoid distribution is described, and the features of patients whose neoplasms have had a sporotrichoid pattern are reviewed.

## Case presentation

An 82-year-old man presented with the new onset of rapidly enlarging skin lesions on his right leg. He has a history of actinic keratosis (periodically treated with cryotherapy using liquid nitrogen) and four non-melanoma skin cancers (three basal cell carcinomas and one squamous cell carcinoma that were excised without recurrence).

**Figure 1:**
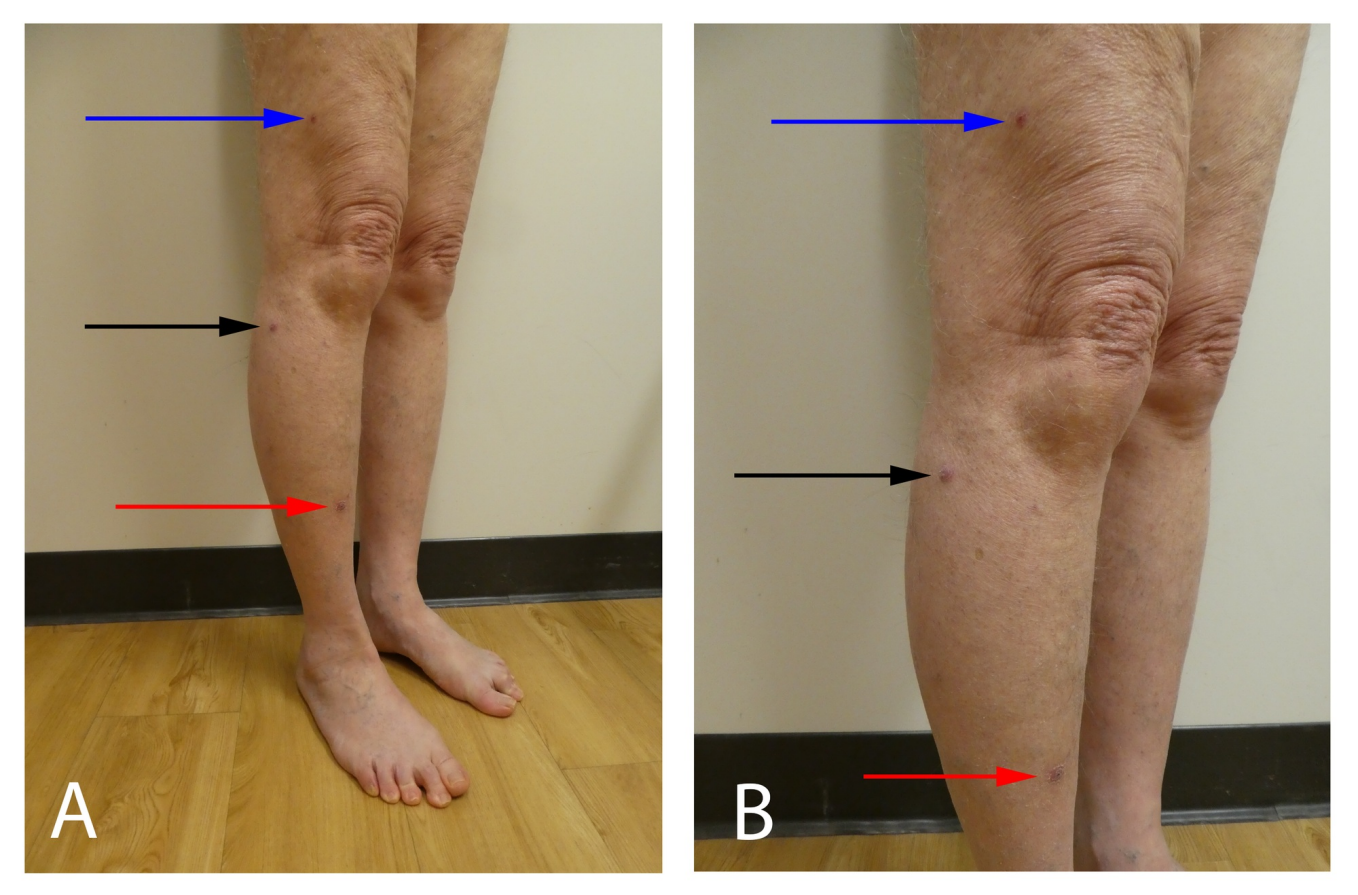
Sporotrichoid keratoacanthomas on the right leg Distal (A) and close (B) views of the right lower leg of an 82-year-old man show three erythematous nodules with peripheral scaling that are present in a sporotrichoid distribution. The distal lesion on the pretibial leg (red arrow) is the largest (1.5 x 1.5 cm) and the proximal lesion on the right thigh (blue arrow) is the smallest (5 x 5 mm); the middle lesion, located inferiolateral to the knee (black arrow), is 1.0 x 1.0 cm.

**Figure 2:**
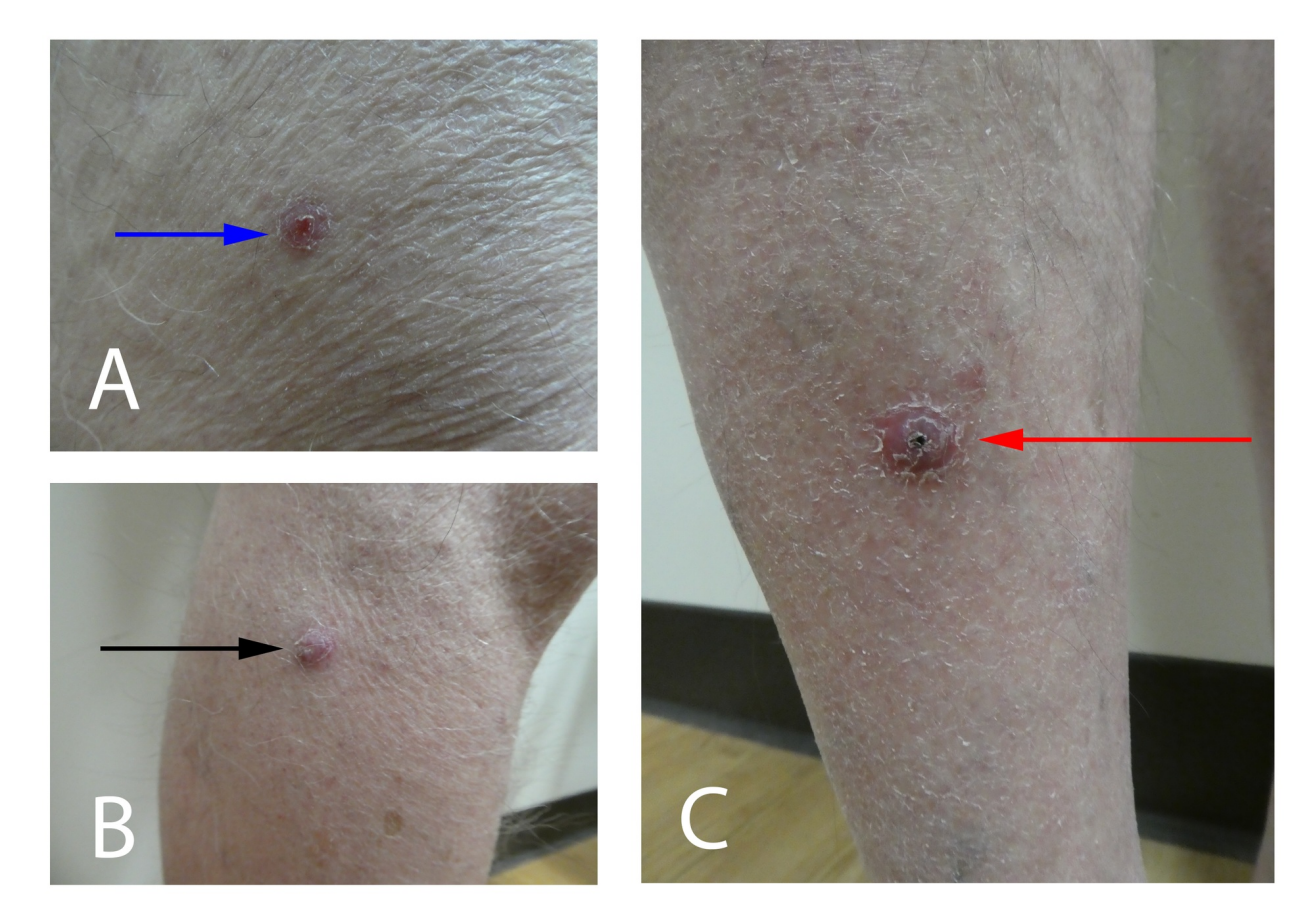
Individual keratoacanthomas that presented in a sporotrichoid pattern The individual keratoacanthomas had a sudden onset and rapid growth. They appeared on the right leg of an 82-year-old man and in a sporotrichoid distribution. The tumors were located on the right distal thigh (A) (blue arrow), the right lateral leg inferior to the knee (B) (black arrow), and the right pretibial leg (C) (red arrow).

Cutaneous examination showed three nontender erythematous nodules that presented in a sporotrichoid pattern, with peripheral scaling and central crust, on his right leg (Figures 1, 2). The proximal lesion was 5 x 5 mm and located on his distal thigh, the distal lesion was 1.5 x 1.5 cm and located on his right pretibial area, and the middle lesion was 1.0 x 1.0 cm and located lateral and inferior to his knee. A biopsy for pathology was performed from all of the lesions; the distal lesion was also bisected and tissue was sent for bacterial, fungal, and mycobacterial cultures.

Microscopic examination of all three lesions showed similar pathologic findings. There was parakeratosis filling a cup-shaped depression in the epithelium. The keratinocytes in the hyperplastic epidermis had glossy-appearing cytoplasm and nuclear atypia. All of the cultures were negative for infectious organisms.

Correlation of the clinical presentation, pathology findings, and laboratory results established a diagnosis of eruptive keratoacanthomas in a sporotrichoid distribution. Evaluation of the groin, axillae, and neck were negative for palpable adenopathy. The lesional sites were excised to ensure complete removal of the tumors. There is no evidence of recurrence at his follow-up examination six months later.

## Discussion

Keratoacanthomas are low-grade, cutaneous neoplasms that present with an initial phase of rapid growth. Although some keratoacanthomas may spontaneously regress, many persist and can continue to enlarge. Therefore, excision of the tumor is often recommended.

Sporotrichosis is caused by the fungus *Sporothrix schenckii*; it can present with pulmonary, cutaneous, or mixed features. Infection is acquired through inhalation from organic material such as soil; however, skin lesions can be acquired by direct inoculation. Lymphocutaneous sporotrichosis is the most common presentation of this disease and often involves nodules that may ulcerate or drain. Treatment is with systemic antifungals.

A PubMed search was performed using the terms cancer, infection, keratoacanthoma, neoplasm, sporotrichoid, sporotrichosis, and tumor; a potential limitation of this paper is that there may be other patients with sporotrichoid conditions that were not discovered based upon the terms we used in our literature search. In addition to sporotrichosis, there are several infectious, neoplastic, and miscellaneous conditions that can present with skin findings that follow cutaneous lymphatics in a sporotrichoid pattern (Table 1) [3-20]. Although *Mycobacterium marinum  *commonly presents in this manner, sporotrichoid skin lesions have not only been observed with other mycobacterial infections but also other bacterial, fungal, protozoal, and viral infections; therefore, in patients presenting with sporotrichoid lesions, tissue should be biopsied not only for histology but also for cultures of bacterial, fungal, and mycobacterial organisms. Rarely, reactive dermatoses, such as Sweet’s syndrome, can present with skin lesions that are distributed in a sporotrichoid manner [11].

**Table 1:** Conditions that can present in a sporotrichoid manner Abbreviations: CR, current report; Ref, reference. ^a^Nocardia includes Nocardia asteroids, Nocardia brasiliensis, and Nocardia nova. ^b^Sporothrix includes Sporothrix brasiliensis and Sporothrix schenckii. ^c^Staphylococcus includes Staphylococcus aureus and Staphylococcus lugdunensis. ^d^Cryptococcus includes Cryptococcus diffluens and Cryptococcus neoformans. ^e^This organism was previously referred to as Pseudallescheria boydii before being renamed. ^f^Mycobacteria include Mycobacteria abscessus, Mycobacteria avium, Mycobacteria celatum, Mycobacteria chelonae, Mycobacteria fortuitum, Mycobacteria haemophilum, Mycobacteria indicus, Mycobacteria marinum, Mycobacteria kansasii, Mycobacteria leprae, Mycobacteria szulgai, and Mycobacteria tuberculosis. ^g^Leishmania includes include Leishmania braziliensis, Leishmania major, Leishmania panamensis, and Leishmania tropica.

Conditions	Ref
Infectious	[3-6]
Bacterial	[3-4]
Bacillus anthracis	[3]
Francisella tularensis	[3]
Nocardia^a^	[3]
Nocardiopsis dassonvillei	[3]
Pseudomonas pseudomallei	[3]
Sporothrix^b^	[3]
Staphylococcus^c^	[3]
Streptococcus pyogenes	[3]
Yersinia pseudotuberculosis	[4]
Fungal	[3-6]
Alternaria infectoria	[3]
Aspergillus nidulans	[3]
Blastomyces dermatitidis	[3]
Coccidioides immitis	[3]
Cryptococcus^d^	[3]
Exophiala polymorpha	[3]
Fonsecaea pedrosoi	[3]
Fusarium solani	[5]
Histoplasma capsulatum	[3]
Paecilomyces lilacinus	[3]
Phialophora verrucosa	[3]
Scedosporium apiospermum^e^	[6]
Taeniolella boppii	[3]
Mycobacterial^f^	[3]
Protozoal	[3]
Acanthamoeba	[3]
Leishmania^g^	[3]
Viral	[3]
Cowpox virus	[3]
Miscellaneous	[7-11]
Calcinosis cutis (iatrogenic)	[7]
Granuloma annular-like dermatitis	[8]
Lupus erythematosus profundus	[9]
Rheumatoid arthritis subcutaneous nodules	[10]
Sweet’s syndrome (acute febrile neutrophilic dermatosis)	[11]
Neoplastic	[12-20]
Epithelioid sarcoma	[17]
Keratoacanthoma	CR
Langerhans cell histiocytosis (adult-onset)	[18]
Lymphoma	[15,16]
B cell	[15]
Primary cutaneous diffuse	[15]
T cell	[16]
Primary cutaneous peripheral, not otherwise specified	[16]
Melanoma	[19]
Metastatic cutaneous	[19]
Peripheral nerve sheath tumor	[20]
Squamous cell carcinoma	[12-14]
Cutaneous, metastatic	[12-14]

Our patient’s three primary keratoacanthomas appeared suddenly; they grew rapidly and were located in a sporotrichoid distribution on his right leg. Other neoplastic processes, albeit rarely, have also been observed to present in a sporotrichoid distribution (Table 2) [12-20]. The associated neoplasms include epithelioid sarcoma (one patient), Langerhans cell histiocytosis (one patient), lymphoma (two patients), melanoma (one patient), peripheral nerve sheath tumor (one patient), and squamous cell carcinoma (three patients).

**Table 2:** Neoplasms presenting with skin lesions in a sporotrichoid distribution Abbreviations: A, age (years); C, case; CR, current report; G, gender; ILK, intralesional kenalog; LLE, left lower extremity; LUE, left upper extremity; M, man; Mult, multiple; Num, numerous; PUVA, psoralen and ultraviolet A; Ref, references; RLE, right lower extremity; RUE, right upper extremity; W, woman; >, greater than; #, number of lesions.

C	A,G	Neoplasm	Site	#	Treatment	Ref
1	63,M	Squamous cell carcinoma (cutaneous, metastatic)	RUE	12	Surgical debulking	[12]
2	70,M	Squamous cell carcinoma (cutaneous, metastatic)	RUE	4	Amputation	[13]
3	83,M	Squamous cell carcinoma (cutaneous, metastatic)	LUE	>5	Systemic chemotherapy, 13-cis-retinoic acid, amputation	[14]
4	50,M	Lymphoma - Primary cutaneous diffuse large B-cell lymphoma, leg type	RLE	5	Radiation	[15]
5	69,M	Lymphoma - Primary cutaneous peripheral T cell lymphoma, not otherwise specified	LUE	3	Radiation, systemic chemotherapy	[16]
6	36,M	Epithelioid sarcoma	LUE	Num	Excision, topical and systemic chemotherapy	[17]
7	82,M	Keratoacanthoma	RLE	3	Excision	CR
8	49,W	Langerhans cell histiocytosis (adult-onset)	LLE	Mult	ILK, systemic chemotherapy, PUVA	[18]
9	28,W	Melanoma (metastatic)	RLE	Mult	Not stated	[19]
10	64,M	Peripheral nerve sheath tumor	RLE	Mult	Amputation, systemic chemotherapy	[20]

Including our patient, 10 individuals have been described whose neoplasms occurred in a sporotrichoid pattern: eight men and two women. The age of the patients at the time of tumor diagnosis ranged from 28 to 83 years (median = 63.5 years). More than half the patients (six of 10 individuals) were older than 60 years.

The tumors either affected the upper extremity (five of 10 individuals) or the lower extremity (five of 10 individuals). Three patients presented with lesions on the left arm while two patients presented with lesions on the right arm. Four patients presented with lesions on the right leg. One patient presented with lesions on the left leg.

Half of the patients (five of 10 individuals) received systemic chemotherapy. Other therapies to treat the neoplastic tumors included amputation (three of 10 individuals), surgical excision (two of 10 individuals), and radiation (two of 10 individuals). Additional treatments were surgical debulking, intralesional kenalog, psoralen and ultraviolet A radiation, 13-cis-retinoic acid, and topical chemotherapy using miltefosine.

To the best of our knowledge, this is the first patient described whose keratoacanthomas presented in a sporotrichoid pattern. Each of his three keratoacanthomas were a primary tumor. Similarly, six of the other patients had primary neoplasms that appeared in a sporotrichoid manner at presentation. However, four of the patients’ tumors represented recurrence and/or metastatic disease presenting with a sporotrichoid distribution of the neoplastic lesions.

Three patients died despite treatment. The 70-year-old man with metastatic cutaneous squamous cell carcinoma had progressive malignant disease following his amputation; he died from a neoplastic lung embolism four months after diagnosis [13]. The 83-year-old man with metastatic cutaneous squamous cell carcinoma also had progressive malignant disease following amputation and systemic therapies; he also died after a thromboembolic event [14]. The 50-year-old man with primary cutaneous diffuse large B-cell lymphoma also had widely disseminated disease to multiple organs; he died of respiratory failure four months after diagnosis [15].

## Conclusions

Infections, neoplasms, and miscellaneous conditions may appear in a sporotrichoid distribution. A man whose eruptive keratoacanthomas presented in a sporotrichoid pattern on his right leg is described. Nine other individuals have also had cancers that have been observed to occur in a sporotrichoid manner; the malignancies include epithelioid sarcoma (one patient), Langerhans cell histiocytosis (one patient), lymphoma (two patients), melanoma (one patient), peripheral nerve sheath tumor (one patient), and squamous cell carcinoma (three patients). The sporotrichoid-appearing neoplasm either presented on the upper extremity (five patients) or lower extremity (five patients). Treatment of the individuals whose neoplasms had sporotrichoid spread included either systemic chemotherapy (five patients) or surgical intervention (six patients) and/or radiotherapy (two patients). Three of the patients with persistent neoplastic disease died from adverse events that may have been malignancy-associated. In summary, keratoacanthoma can be added to the list of cancers having cutaneous lesions presenting in a sporotrichoid distribution. In addition, biopsy of new skin lesions—not only for microscopic examination but also for bacterial, fungal, and mycobacterial cultures—should be considered when they appear in sporotrichoid manner.
